# A Contrastive Domain Adaptation Framework for Knee Osteoarthritis Severity Grading

**DOI:** 10.3390/bioengineering13090975

**Published:** 2026-08-25

**Authors:** Weiqiang Liu, Minghui Wu, Keming Liu, Mingyao Wu, Yunfeng Wu

**Affiliations:** 1 School of Computer Science, Minnan Normal University, Zhangzhou 363000, China; lwq2688@mnnu.edu.cn (W.L.); 16627520673@163.com (M.W.); lkm2407039626@163.com (K.L.); wmy18150784981@163.com (M.W.); 2 Key Laboratory of Data Science and Intelligence Application, Fujian Province University, Zhangzhou 363000, China; 3 Research Institute of Embodied Interaction Science and Technology, Minnan Normal University, Zhangzhou 363000, China; 4 School of Informatics, Xiamen University, 422 Siming South Road, Xiamen 361005, China

**Keywords:** knee osteoarthritis, Kellgren–Lawrencegrading, knee X-ray, contrastive learning, domain adaptation

## Abstract

Kneeosteoarthritis (KOA) is a common degenerative joint disease that causes pain, stiffness, and impaired mobility. Automated Kellgren–Lawrence (KL) grading from knee X-ray images facilitates efficient screening and follow-up assessment. However, models trained on a single-source dataset frequently suffer performance degradation when applied to external cohorts, due to heterogeneities in image quality, acquisition protocols, class distributions, and annotation patterns. Furthermore, conventional domain adaptation approaches typically treat all source samples uniformly, making them vulnerable to negative transfer induced by ambiguous or distributionally divergent instances. To overcome these limitations, the present study develops a supervised contrastive domain adaptation framework designed for robust KOA severity grading under domain shift. The framework incorporates two task-specific modules: (1) a source-domain sample screening module that dynamically allocates class-wise quotas based on transferability and identifies high-value source samples by evaluating target intra-class affinity, inter-class separability, and source-class compactness; and (2) a target-balanced ordinal contrastive learning module that aligns the screened source samples with target features and imposes stronger constraints on negative pairs with larger KL-grade distances. The framework was evaluated bidirectionally on KneeKL (8260 images) and MedicalExpert-I (1650 images), two public knee radiograph datasets for KOA grading. With ResNet-18, it achieved a Quadratic Weighted Kappa (QWK) of 0.8557 for KneeKL-to-MedicalExpert-I transfer, exceeding source-only training and direct source–target merging by 0.2652 and 0.0468, respectively. Comparisons with representative existing methods and multiple experimental analyses further validate the competitiveness of the proposed framework.

## 1. Introduction

The knee joint is the largest synovial joint in the human body and plays a central role in weight bearing, shock absorption, and locomotion. Its stability relies on the coordinated function of the tibiofemoral and patellofemoral compartments, menisci, ligaments, cartilage, and surrounding soft tissues for weight bearing and locomotion [1]. Degeneration of these structures may lead to knee osteoarthritis (KOA), a chronic disease commonly associated with pain, stiffness, reduced range of motion, and reduced daily activity [2]. Recent epidemiological studies indicate an increasing global burden of osteoarthritis, with knee involvement remaining a primary contributor [3]. Factors such as high body mass index, aging, prior knee trauma, and long-term mechanical loading further exacerbate the risk of KOA and are expected to contribute to a continued increase in disease prevalence in the coming decades [4].

Despite the diagnostic sensitivity of magnetic resonance imaging (MRI), plain radiography remains the gold standard for KOA evaluation due to its cost-effectiveness, accessibility, and compatibility with established clinical grading systems [5]. The Kellgren–Lawrence (KL) scale, which categorizes severity from 0 to 4 based on structural features like osteophytes, joint-space narrowing, sclerosis, and bony deformity, is the most widely adopted metric [6]. Although X-ray imaging is less sensitive to early soft-tissue pathology than MRI, it remains indispensable in routine screening and clinical monitoring [7]. Consequently, the present study focuses on five-class KL grading from plain knee radiographs, which remains a clinically relevant and widely available imaging setting.

Recent advances in deep learning have substantially improved automated KOA grading [8]. Convolutional neural networks (CNNs) learn hierarchical image representations through stacked convolutional layers, progressively transforming low-level patterns such as edges and textures into higher-level structural features. This hierarchical representation is well suited to radiographic KOA grading, where disease severity is characterized by structural changes such as osteophyte formation, joint-space narrowing, sclerosis, and bony deformation. Recent reviews have shown that KL-based X-ray classification remains one of the main tasks in automated KOA assessment [9]. However, the generalizability of these models is frequently compromised by domain shift. Knee radiographic data often exhibit substantial heterogeneity across institutions in terms of image resolution, exposure, acquisition protocol, preprocessing, annotation practice, and patient composition [10]. Such distributional discrepancies constitute a fundamental barrier that causes a model trained on a source domain to suffer performance degradation when applied to a target domain [11].

This challenge is compounded by two task-specific characteristics of KL grading. First, the KL grading system is inherently ordinal: misclassifying KL0 with KL1 is clinically less severe than confusing KL0 with KL4 [12]. Second, datasets typically exhibit class imbalance, with mild grades usually more common than severe grades [13]. Ordinal loss has been used to reduce grade-distance errors, but it does not by itself solve cross-domain feature mismatch [14]. Furthermore, conventional domain adaptation methods typically align source and target features using the entire source dataset. This may be suboptimal for KOA grading because ambiguous, noisy, or distribution-divergent source samples can induce negative transfer in the target feature space. A recent musculoskeletal adaptation study also suggests that strategic source-domain sample screening can be useful when contrastive learning is used across domains [15].

To address these limitations, we developed a framework to incorporate a source-domain sample screening strategy and a target-balanced ordinal contrastive learning module for radiographic KOA grading. KneeKL and MedicalExpert-I are two publicly available knee radiograph datasets covering all five KL grades. KneeKL is derived from the Knee Osteoarthritis Severity Grading Dataset [16], whereas MedicalExpert-I is a subset of the Digital Knee X-ray Images dataset [17]; detailed experimental splits and class distributions are provided in Section 4.1. The proposed method uses KneeKL and MedicalExpert-I as two domains to evaluate bidirectional transfer. Instead of aligning all source data samples, our method screens source-domain samples with higher transfer value. The selection process integrates class-level difficulty-based quota allocation, prototype-based sample quality scoring, and a core/adapt sampling strategy. The screened source samples are then aligned with target-domain features through a target-balanced ordinal contrastive loss. This design aims to preserve five-class discriminability, mitigate the influence of unreliable source samples, and maintain the ordinal structure of KL grades.

The primary contributions of the present work are summarized as follows:

1. This study investigates supervised domain adaptation for five-class KOA severity grading using the KneeKL and MedicalExpert-I, and it evaluates bidirectional transfer performance across two representative CNN backbones.
2. A source-domain sample screening strategy is developed to select transfer-informative source samples before contrastive alignment, where class-wise quotas, prototype-based quality scoring, and core/adapt sampling are jointly considered.
3. This study is the first to introduce source–target contrastive learning into supervised domain adaptation for radiographic KOA KL grading. Specifically, the proposed loss aligns screened source-domain samples with the target-domain feature bank while considering class imbalance and KL-grade distance. Our experimental results support the effectiveness of the proposed framework.

## 2. Related Work

### 2.1. Radiographic KOA Severity Grading

The field of automated KOA grading from radiographs has undergone a significant paradigm shift from hand-crafted feature analysis toward advanced deep learning-based prediction. Antony et al. [18] framed severity estimation as a learning problem and demonstrated that convolutional networks could improve KL-grade prediction from knee X-rays. Tiulpin et al. [19] later proposed a Siamese network that exploits bilateral knee information to help achieve robust diagnostic performance on plain radiographs. Thomas et al. [20] developed an end-to-end network for radiographic KOA severity classification and reported inter-rater agreement levels comparable to those of expert clinicians.

More broadly, recent reviews of region-based CNNs have highlighted both the strengths and limitations of convolutional architectures in complex visual recognition. Sastra et al. [21] reported that feature fusion, attention mechanisms, and multi-scale representations improve Faster R-CNN in challenging scenarios such as occlusion and small-object detection, while computational complexity, inference latency, and data dependence remain important limitations. Similarly, Erniwati et al. [22] showed that data augmentation and enhanced feature extraction can improve the robustness of Mask R-CNN, although high computational cost and sensitivity to data scarcity and environmental complexity remain challenges. Although these studies focus on object detection and instance segmentation rather than KL grading, they illustrate the representational strengths and practical trade-offs of CNN-based methods.

Recently, the research focus has pivoted toward external validation and real-world clinical deployment. Vaattovaara et al. [23] evaluated a deep learning-based KL grading model on an independent external dataset by comparing its performance against multiple human readers and underscoring the critical necessity of out-of-distribution testing. Similarly, Nasef et al. [24] investigated automated KL grading across two distinct public knee X-ray datasets; they found that model performance could be highly sensitive to variations in dataset characteristics and architectural choices. These findings strongly motivate our work: the clinical utility of radiographic KOA grading models cannot be assessed solely through internal dataset accuracy, as the real-world deployment frequently exposes algorithms to heterogeneous data distributions.

In spite of these notable advancements, several limitations remain. First, many existing approaches still rely on conventional classification loss functions, and they may fail to fully exploit the inherent ordinal nature of the KL grading scale. Second, class imbalance remains a pervasive challenge in KOA datasets, and it commonly leads to model optimization bias toward majority classes [25]. Moreover, indiscriminate utilization of source-domain samples during adaptation can introduce poorly matched or ambiguous instances and can increase the risk of negative transfer [26]. To address these challenges, the present study proposes a supervised contrastive domain adaptation framework that integrates source-domain sample screening with ordinal contrastive alignment.

### 2.2. Feature Transfer and Domain Adaptation

Given the scarcity of annotated medical data, transfer learning has become a ubiquitous paradigm in medical imaging. ImageNet-pre-trained models remain common in medical image analysis, even though source and medical target domains differ substantially in texture, anatomy, and visual semantics [27]. From a feature-transfer perspective, lower network layers typically capture general low-level features whereas higher layers become increasingly task-specific and are more difficult to transfer [28]. This dichotomy explains the efficacy of fine-tuning, while also elucidating why a model trained on one radiographic dataset may still suffer performance degradation when applied to another.

Feature alignment methods aim to bridge the distributional gap between source and target domains. Representative approaches, such as Maximum Mean Discrepancy (MMD)-based adaptation and correlation alignment, intend to match global feature statistics across domains [29,30]. These methods are straightforward and typically treat all source samples with equal weights. Adversarial adaptation offers an alternative strategy by training a feature extractor to fool a domain discriminator, with Domain-Adversarial Neural Network (DANN) and Adversarial Discriminative Domain Adaptation (ADDA) serving as classic examples [31,32]. Although adversarial learning can strengthen domain invariance, it is often plagued by training instability. Furthermore, the indiscriminate inclusion of poor-quality source samples during alignment can still precipitate negative transfer [33].

Contrastive learning provides a distinct mechanism for structuring the feature space. Objectives such as InfoNCE and supervised contrastive learning operate by pulling similar samples closer together while pushing dissimilar ones apart [34,35]. For KL grading, however, standard contrastive learning exhibits a critical limitation: it treats all samples from different grades as equally negative, and it also disregards the inherent ordinal nature of clinical severity [36]. To address this, our work adapts contrastive learning for radiographic KOA grading by integrating source-domain sample screening, balancing the contribution of each target-domain class and weighting negative pairs according to their KL grade distance.

## 3. Proposed Method

### 3.1. Overall Framework

The proposed framework addresses the task of supervised domain adaptation for KL grading of KOA, and it is formulated under the assumption that labeled samples from the target domain are available during the adaptation phase. Such a task presents distinct challenges compared with conventional image classification, in two important aspects. First, source and target datasets frequently may vary in imaging protocols, image quality, patient demographics, and class distributions. Second, KL grading labels possess an inherent ordinal nature, with progressive disease severity gradually increasing from grade 0 (healthy) to grade 4 (severe). Consequently, an effective model must simultaneously satisfy three objectives: preserving discriminative power across all five severity classes, mitigating the risk of negative transfer induced by source samples with low transferability, and rigorously maintaining the ordinal severity relationships among KL grades within the learned feature space.

Let the source-domain training set be $D_{s}=\left\{\left(x_{i}^{s},y_{i}^{s}\right)\right\}$ and the target-domain training set be $D_{t}=\left\{\left(x_{j}^{t},y_{j}^{t}\right)\right\}$, where y∈{0,1,2,3,4} denotes the five ordered severity grades from KL 0 to KL 4. Given a knee X-ray image *x*, the classification network consists of a feature encoder h(·) and a classification head g(·). The encoder extracts the deep feature $z_{i}=h\left(x_{i}\right)$, and the classification head predicts the class probability $p_{i}=\mathrm{softmax}\left(g\left(z_{i}\right)\right)$ over the five KL grades. The feature $z_{i}$ is then used for subsequent source-sample screening and cross-domain contrastive learning.

The proposed method consists of three functional modules: a baseline KL grading network, a source-domain sample screening module, and a contrastive learning module. The baseline KL grading network provides sample-level class probabilities and deep image features. The source-domain sample screening module selects source samples with higher transfer value from the source-domain training set, avoiding the indiscriminate inclusion of all source samples in cross-domain alignment. The contrastive learning module uses a labeled target-domain feature bank to establish an ordinal contrastive relationship between source samples and the target-domain class structure. Unlike methods that rely only on merged training or global distribution alignment, our method jointly considers which source samples should participate in feature alignment and how they should be aligned according to the target-domain class structure, and it optimizes these components through a unified training objective.

**Figure 1:**
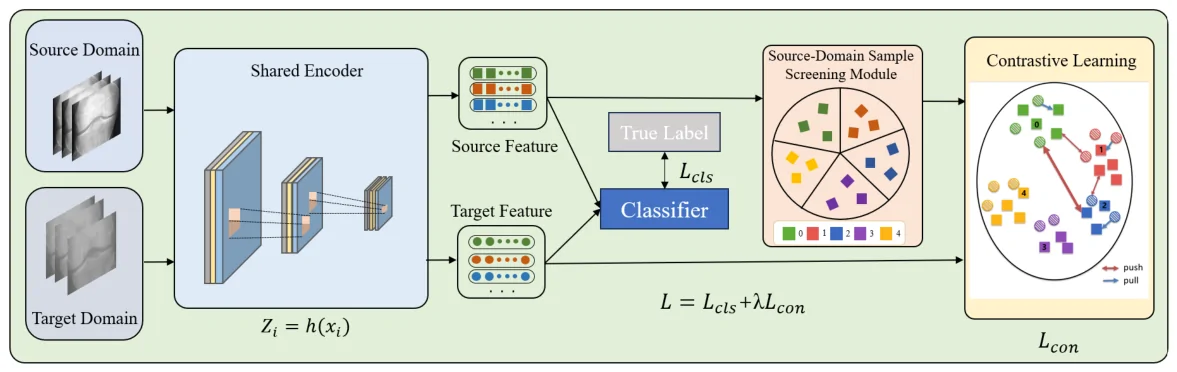
Overall framework of the proposed supervised contrastive domain adaptation method for knee osteoarthritis (KOA) Kellgren–Lawrence (KL) grading. Colors indicate different KL-grade categories, while squares and shaded circles represent source-domain and target-domain samples, respectively. Solid arrows indicate the main processing flow through the framework.

The overall workflow of the proposed framework is illustrated in Figure 1. The framework first extracts deep features and class probabilities from labeled source- and target-domain images. Then, the source-domain sample screening module selects transfer-informative source samples according to class-level difficulty and prototype-based quality. Finally, the selected source samples are aligned with the target-domain feature bank through target-balanced ordinal contrastive learning.

### 3.2. Source-Domain Sample Screening Module

**Figure 2:**
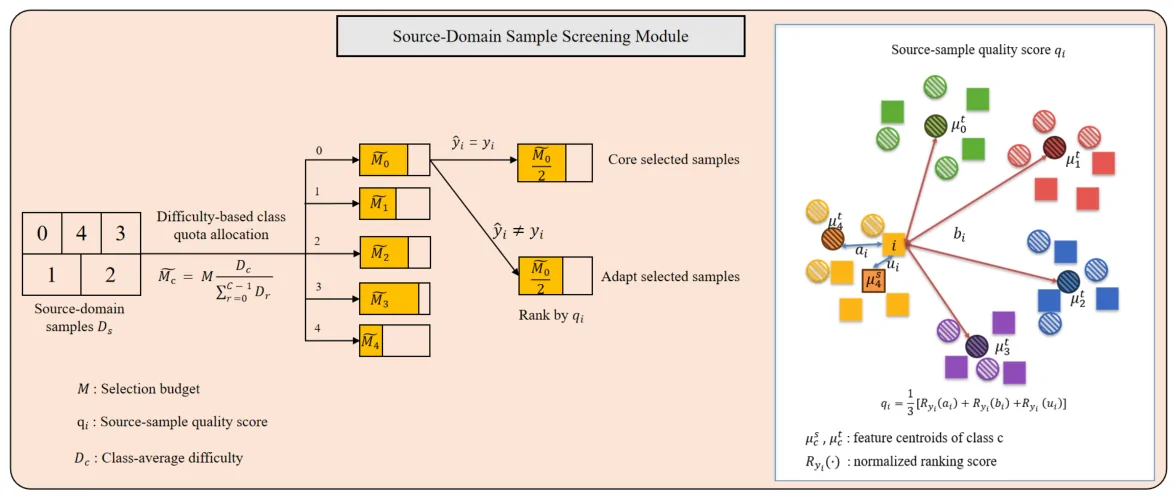
Source-domain sample screening module. Class-average difficulty is first used to allocate class-wise selection quotas. Within each KL grade, source samples are then scored according to target same-class affinity, target cross-class separation, and source-class representativeness. Correctly predicted and misclassified candidates are sorted by the quality score and selected from the core/adapt pools to form the final selected source set $A_{s}$. In the lower-right schematic, source-domain and target-domain samples are represented by squares and circles, respectively; the class centroids are shown in a darker color and identified by their corresponding symbols.

The source-domain sample screening module aims to select transferable source samples from the source-domain training set rather than simply treating all source samples as cross-domain alignment objects. For KOA KL grading, different datasets may differ in imaging style, acquisition protocol, class distribution, and annotation boundaries. Some source samples may be useful within the source domain but may not be consistent with the target-domain structure of the same KL grade. If such samples directly participate in contrastive learning then they may pull target-domain features toward unstable or incorrect regions. Therefore, the source-sample screening process is organized into three consecutive steps: first, allocating selection quotas according to class-level transfer difficulty; second, computing sample transfer quality according to source and target prototypes; and third, constructing a mixed core/adapt source-sample set within each class. The detailed procedure of the source-domain sample screening module is shown in Figure 2:

First, the candidate quotas for each KL grade are allocated according to the average source-domain class difficulty. Let ${\hat{y}}_{i}$ be the current predicted grade of source sample $x_{i}^{s}$, and let $p_{i,y_{i}}$ be the model-predicted probability of the true class $y_{i}$. The difficulty of a source sample is defined as

(1) $$d_{i}=\left(1-p_{i,y_{i}}\right)+\frac{|{\hat{y}}_{i}-y_{i}|}{C-1},$$

where C=5. The first term reflects the model uncertainty with respect to the true class, while the second term reflects the ordinal distance between the predicted grade and the true grade. Compared with using classification confidence alone this definition distinguishes adjacent-grade misclassification from long-range grade misclassification, making the source-sample difficulty more consistent with the ordinal nature of KL grading.

After obtaining the sample difficulty, the average difficulty of each class is computed as

(2) $$D_{c}=\frac{1}{N_{c}^{s}}\sum_{i:y_{i}=c}d_{i},$$

where $N_{c}^{s}$ denotes the number of source-domain samples in class *c*. A higher class-average difficulty indicates that the current model has greater discriminative uncertainty and ordinal error for that grade. Thus, that class should be given more opportunities for selection and cross-domain alignment.

Let the total selection budget be *M*. The continuous candidate quota for class *c* is allocated according to the average difficulty and is then integer-adjusted while keeping the total budget unchanged:

(3) $${\tilde{M}}_{c}=M\frac{D_{c}}{\sum_{r=0}^{C-1}D_{r}}.$$

This allocation strategy prevents the candidate set from being dominated by majority classes in the source domain while also avoiding the limitation of uniform allocation, which ignores differences in class-level transfer difficulty. It should be emphasized that class-average difficulty is used only to allocate the candidate quota for each KL grade; it does not mean that the most difficult samples are directly selected. The source samples that actually enter contrastive learning still need to be evaluated within each class by prototype-based quality scoring. This separates class coverage from sample reliability, allowing the selection process to focus on difficult grades while avoiding the mistaken selection of noisy samples as high-value samples.

After determining the selection quota for each class, source-sample quality is further evaluated using source-domain and target-domain class centroids. Let $\mu_{c}^{t}$ and $\mu_{c}^{s}$ denote the feature centroids of class *c* in the target domain and source domain, respectively. For a source sample *i*, its transfer value is measured from three aspects: its consistency with the target-domain centroid of the same class, denoted by $a_{i}$; its separation from target-domain centroids of other classes, denoted by $b_{i}$; and its representativeness within the source-domain same-class structure, denoted by $u_{i}$. Let $R_{c}\left(\cdot \right)$ denote the normalized ranking score obtained by sorting values in descending order within class *c*. The largest value in each class receives the highest normalized score, and the remaining samples are linearly scaled according to their ranks. The source-sample quality score is defined as

(4) $$q_{i}=\frac{1}{3}\left[R_{y_{i}}\left(a_{i}\right)+R_{y_{i}}\left(b_{i}\right)+R_{y_{i}}\left(u_{i}\right)\right],$$

where

(5) $$a_{i}=z_{i}^{⊤}\mu_{y_{i}}^{t},\;b_{i}=-\frac{1}{C-1}\sum_{c\neq y_{i}}z_{i}^{⊤}\mu_{c}^{t},\;u_{i}=z_{i}^{⊤}\mu_{y_{i}}^{s}.$$

A higher quality score indicates that the source sample is closer to the same-grade target-domain structure, farther from other-grade target-domain structures, and still representative of its source-domain class. Therefore, such a sample is more suitable for use as a source-domain sample in cross-domain alignment.

Finally, a mixed core/adapt source-sample set is constructed within each class. Core samples are source samples whose current predicted grade is consistent with the true grade, and they usually provide a more stable same-grade structure. Adapt samples are source samples whose current predicted grade is inconsistent with the true grade but whose quality scores are high; these samples usually contain more obvious boundary or domain-shift information. When selecting core and adapt samples, candidates in each pool are sorted in descending order by $q_{i}$, and the highest-ranked samples are selected first. If one candidate pool does not contain enough samples then the remaining quota for that class is filled by samples from the other pool, still in descending order by $q_{i}$. The final selected source-sample set is denoted as $A_{s}$. This set does not imply that all selected samples are fixed anchors in an absolute sense; instead, it indicates that these source samples are more suitable for contrastive alignment under the current target-domain structure.

### 3.3. Contrastive Learning Module

The contrastive learning module aligns transfer-informative source samples with the target-domain class structure. Compared with constructing positive and negative samples only within a mini-batch the target-domain feature bank preserves a more complete target-domain sample-level distribution, allowing each source-side reference sample to establish relationships with more sufficient same-grade and different-grade target samples. We first construct a target-domain feature bank $B_{t}=\left\{\left(z_{j}^{t},y_{j}^{t}\right)\right\}$, where $z_{j}^{t}$ is the deep feature of a target-domain training sample and $y_{j}^{t}$ is its ground-truth KL label. Positive and negative relationships are determined by the ground-truth KL grades: if $y_{i}=y_{j}$ then target sample *j* is treated as a positive sample for source-side reference sample *i*; otherwise, it is treated as a negative sample. The positive sample set for source-side reference sample *i* is denoted as P(i). The feature similarity between source-side reference sample *i* and target-bank sample *j* is measured by cosine similarity and denoted as $s_{ij}$.

Inspired by InfoNCE [34], supervised contrastive learning [35], and cross-domain contrastive learning in musculoskeletal imaging [15], we introduce KL-grade distance into the contrastive relationship. The motivation is that the difference between KL0 and KL1 should not be treated as equivalent to the difference between KL0 and KL4. Thus, negative samples from adjacent grades should be separated more mildly, whereas negative samples from distant grades should receive stronger separation constraints. The ordinal distance weight is defined as

(6) $$r_{ij}=\left\{\begin{array}{cc} 1, & y_{i}=y_{j},\\ \alpha +\left(1-\alpha \right)\frac{|y_{i}-y_{j}|}{C-1}, & y_{i}\neq y_{j}, \end{array}\right.$$

where C=5 is the number of KL grades. The parameter α controls the baseline separation strength for different-grade negative pairs, which prevents adjacent-grade negatives from being assigned excessively weak constraints while still preserving stronger penalties for larger KL-grade distances. This design keeps the aggregation of same-grade samples unchanged while encoding the ordered severity distance through negative pairs.

We further improve this ordinal contrastive formulation by introducing target-domain balanced weighting. In KOA grading, the target-domain class distribution is usually uneven, and samples from more frequent grades may dominate the denominator of the contrastive loss. Let $n_{c}^{t}$ be the number of target-domain training samples of class *c* in the feature bank. The class-balancing weight for target sample *j* is defined as

(7) $$b_{j}=\frac{\frac{1}{n_{y_{j}}^{t}}}{\frac{1}{C}\sum_{c=0}^{C-1}\left(\frac{1}{n_{c}^{t}}\right)}.$$

This weight increases the contribution of under-represented target grades, while the mean normalization keeps the overall loss scale stable.

Finally, the target-domain class-balancing weight and the KL ordinal distance weight are jointly incorporated into the contrastive logits. The target-balanced ordinal contrastive loss for source-side reference sample *i* is formulated as

(8) $$L_{i}^{\mathrm{con}}=-\frac{1}{\left|P(i)\right|}\sum_{p\in P(i)}\log\frac{\exp\left(s_{ip}+\log\left(b_{p}r_{ip}\right)\right)}{\sum_{k\in B_{t}}\exp\left(s_{ik}+\log\left(b_{k}r_{ik}\right)\right)}.$$

The ordinal distance weight models the severity distance among KL grades, while the target-domain balancing weight reduces the dominance of frequent target grades in contrastive learning. Together, these two components encourage each selected source sample to move closer to target-domain samples of the same grade while preserving a more reasonable ordinal feature structure. Because the contrastive loss is applied only to the selected source-sample set, source samples with low transfer value do not indiscriminately distort the learned feature geometry, thereby reducing the risk of negative transfer.

### 3.4. Total Loss and Optimization

The total loss consists of classification supervision and target-balanced ordinal contrastive supervision. Classification supervision is used to maintain five-class decision boundaries for source-domain and target-domain samples. Specifically, we use the cross-entropy loss and combine it with an EMD auxiliary term used in previous KOA ordinal grading work [14]:

(9) $$L_{\mathrm{cls}}=\mathrm{CE}\left(p,y\right)+\lambda_{\mathrm{emd}}L_{\mathrm{EMD}}\left(p,y\right).$$

Here, the auxiliary coefficient is set to 0.1. The cross-entropy term provides basic category-discrimination supervision, while the EMD term constrains the ordinal structure of KL grades at the probability-distribution level, so that long-range grade errors receive penalties that are more consistent with the grading task. The classification loss ensures that the model maintains stable five-class decision boundaries on labeled source-domain and target-domain samples. The contrastive supervision is computed on the transferable source-sample set $A_{s}$ obtained by the source-domain sample screening module, thereby achieving effective cross-domain alignment.

The final training loss is

(10) $$L=L_{\mathrm{cls}}\left(D_{s},D_{t}\right)+\lambda_{\mathrm{con}}\frac{1}{|A_{s}|}\sum_{i\in A_{s}}L_{i}^{\mathrm{con}}.$$

The complete training procedure of the proposed framework is summarized in Algorithm 1.
**Algorithm 1** Target-Balanced Ordinal Contrastive Learning with Source-Domain Sample Screening**Require:** Source set $D_{s}$, labeled target set $D_{t}$, KL classes C=5, source selection budget *M* **Ensure:** Trained KL grading model with encoder h(·) and classifier g(·)
 1:Initialize the KL grading model 2:**for** each epoch **do** 3:    Forward source and target training images through the current encoder and classifier to obtain logits and features 4:    Compute the classification loss on labeled source and target samples using Equation (9) 5:    Update the model with the classification loss 6:    Build the target feature bank $B_{t}$ and update source/target class prototypes 7:    Estimate source-sample difficulty and allocate class quotas using Equations (1)–(3) 8:    Compute prototype-based source quality scores using Equations (4) and (5) 9:    Select top-ranked *core*/*adapt* samples in each class to form $A_{s}$10:    Determine target positives and negatives for each selected source sample by KL labels11:    Compute ordinal distance weights and target-balanced weights using Equations (6) and (7)12:    Compute contrastive loss on $A_{s}$ using Equation (8)13:    Update the model with the contrastive loss14:**end for**15:**return** Trained encoder h(·) and classifier g(·)


## 4. Experiments and Results

### 4.1. Datasets

The experiments were conducted on two knee radiograph datasets for five-class Kellgren–Lawrence (KL) grading, where the labels correspond to KL0–KL4. The two datasets were treated as different domains to evaluate domain adaptation performance. Their training, validation, and test splits were kept fixed across all the experiments, and the detailed class distributions are shown in Table 1.

#### 4.1.1. KneeKL Dataset

The first dataset utilized in the experiments was obtained from the KL-grading subset of the Knee Osteoarthritis Severity Grading Dataset [16]. The full dataset was released on Mendeley Data and contains knee X-ray images organized from the Osteoarthritis Initiative (OAI) cohort for knee joint detection and KL severity grading. In the experiments, the KL-grading subset was denoted as KneeKL. It contains 5778 training images, 826 validation images, and 1656 test images. The dataset covers all five KL grades but the numbers of images vary across grades, which makes it useful for evaluating model robustness under uneven class distributions.

#### 4.1.2. MedicalExpert-I Dataset

The second dataset utilized in the experiments was the MedicalExpert-I subset of the Digital Knee X-ray Images dataset [17]. The complete dataset is organized into two expert-labeled subsets, namely MedicalExpert-I and MedicalExpert-II, both following the KL grading system for knee osteoarthritis severity. The experiments used the MedicalExpert-I subset because it provides the same five-grade label space as KneeKL while representing a different data domain. In the project split, MedicalExpert-I contains 1152 training images, 245 validation images, and 253 test images. Compared with KneeKL, MedicalExpert-I is smaller, but it provides a complementary domain for evaluating cross-dataset adaptation.

### 4.2. Experimental Design and Evaluation Metrics

#### 4.2.1. Experimental Design

Two domain adaptation settings were evaluated to examine the robustness of KL grading under domain shift. In the KneeKL-to-MedicalExpert-I setting, denoted as K→M, KneeKL was used as the source domain and MedicalExpert-I was used as the target domain. In the reverse setting, denoted as $M_{\mathrm{aug}7}\;\to \;K$, MedicalExpert-I was used to construct the source domain and KneeKL was used as the target domain. Since the original MedicalExpert-I training split is much smaller than KneeKL, the MedicalExpert-I training images were expanded sevenfold in the reverse setting using the same training-time image operations, including random rotation, horizontal flipping, and slight scaling or translation. This produced 8064 source training images for $M_{\mathrm{aug}7}\;\to \;K$. The expansion was applied only to the MedicalExpert-I training split, while validation and test images remained unchanged.

ResNet18 and VGG19 with ImageNet-pre-trained weights were selected as two representative CNN backbones [37,38]. ResNet18 uses residual shortcut connections to facilitate deep-network optimization, whereas VGG19 adopts a conventional sequential architecture based on stacked small convolutional filters. Their distinct architectures allowed the proposed adaptation framework to be evaluated across different CNN backbones. For both networks, the final classifier was replaced with a five-class output layer, and a Softmax function was applied to the classifier outputs to obtain the class probabilities, while the pre-classifier feature vector was directly used for contrastive learning. All images were resized to 224×224, and validation and testing used only re-sizing and normalization.

Three methods were compared under the same backbone: source-only training, direct source–target merging, and the proposed method. The source-only baseline was trained exclusively on the source-domain training split, selected according to source-domain validation performance, and evaluated on the target-domain test split to assess direct cross-domain generalization. The merge baseline jointly trained the model using source- and target-domain training images, selected the model based on the target-domain validation split, and served as a supervised adaptation baseline. Following the same supervised adaptation setting as the merge baseline, the proposed method further introduced transfer-informative source-sample screening and target-balanced ordinal contrastive learning before final evaluation on the target-domain test split.

In addition to the source-only and direct source–target merging baselines, four representative existing methods were introduced for comparison with the proposed framework. These methods were selected to cover three related research directions: ordinal learning, general domain adaptation, and medical image domain adaptation. For ordinal learning, CORN [39] was selected as a representative ordinal regression method, which models ordered labels by decomposing ordinal prediction into a sequence of binary classification problems. CLOC [36] was included as a recent ordinal contrastive learning method that introduces a multi-margin N-pair contrastive objective to preserve ordinal relationships in feature space. For domain adaptation, DANN [31] was selected as a representative adversarial adaptation method. In addition, SAT-DA [40] was included as a recent supervised medical domain adaptation method. Since DANN was originally developed under an unsupervised domain adaptation setting, target-domain classification supervision was additionally incorporated to adapt it to the supervised domain adaptation protocol adopted in this study while preserving its original adaptation mechanism. CORN and CLOC were originally designed for single-domain ordinal classification; therefore, labeled source and target-domain samples were jointly utilized as supervised ordinal baselines. For each competing method, necessary modifications related to dataset format, number of classes, and evaluation protocol were introduced to enable comparison on the KOA grading task.

All the experiments were conducted on a Linux workstation equipped with an NVIDIA GeForce RTX 4090 D GPU (NVIDIA Corporation, Santa Clara, CA, USA). The implementation was based on Python 3.10.19, PyTorch 1.13.1 with CUDA 11.6, and torchvision 0.14.1. All figures and visualizations were generated using Matplotlib 3.10.6 with NumPy 2.3.5. The batch size was set to 32, and Adam was used for adaptation training with a learning rate of $5\times 10^{-6}$. The source sample retention ratio was set to ρ=0.1, the core/adapt ratio in source-sample screening was set to 0.5, and the ordinal distance parameter was set to α=0.25. The ordinal EMD weight in the classification objective was set to 0.1. The final results were obtained with random seed 1118. Early stopping was performed according to the validation QWK to ensure that model selection was consistent with the primary ordinal grading metric. Since different backbones showed different convergence behaviors in preliminary experiments, the patience was set to 10 epochs for ResNet18 and 15 epochs for VGG19. Importantly, for the Source-only, Merge, and proposed methods evaluated under the same backbone, the same early-stopping criterion and patience value were used, and the independent test set was used only once for final performance reporting.

#### 4.2.2. Evaluation Metrics

The primary evaluation metric was quadratic weighted kappa (QWK), which is widely used for ordinal grading tasks because it assigns larger penalties to predictions farther away from the ground-truth grade. Let *O* and *E* denote the observed and expected confusion matrices, respectively. QWK is computed as

(11) $$\mathrm{QWK}=1-\frac{\sum_{i=0}^{C-1}\sum_{j=0}^{C-1}w_{ij}O_{ij}}{\sum_{i=0}^{C-1}\sum_{j=0}^{C-1}w_{ij}E_{ij}},\;w_{ij}=\frac{{\left(i-j\right)}^{2}}{{\left(C-1\right)}^{2}},$$

where C=5 is the number of KL grades. A higher QWK indicates better agreement between model predictions and ground-truth KL labels while accounting for the ordinal distance between grades.

Mean absolute error (MAE) was used to measure the average ordinal distance between predicted and ground-truth KL grades. A lower MAE indicates fewer large-distance grading errors.

Accuracy (ACC) was calculated as the proportion of correctly classified testing images among all the testing images. It directly reflected the overall classification correctness but did not distinguish between adjacent-grade and distant-grade errors.

Macro-F1 was calculated by first computing the F1 score independently for each KL grade and then averaging the five class-wise F1 scores with equal weight. This metric gives the same importance to each KL grade and is therefore useful when class frequencies are uneven.

Balanced accuracy (BACC) was calculated as the average recall across the five KL grades. It measured whether the model maintained stable sensitivity for different severity levels rather than performing well only on classes with more samples.

### 4.3. Analysis of Experimental Results

#### 4.3.1. Performance Comparison

Table 2 and Table 3 present the performance results on the target-domain testing set under four transfer settings. The comparison includes source-only training, direct source–target merging, and the proposed method. The QWK was used as the primary metric to account for the ordinal nature of KL grading. In addition, ACC, MAE, Macro-F1, and BACC were utilized to provide a complementary evaluation of overall correctness, grading error, and class-balanced performance. Bold values indicate the best-performing result(s) within the corresponding comparison, while ↑ and ↓ indicate that higher and lower values are better, respectively. These conventions apply to Table 2, Table 3, Table 4, Table 5, Table 6 and Table 7.

The source-only results were consistently inferior to the adapted methods, which demonstrated that directly transferring a model trained on one radiograph dataset to another results in substantial performance degradation. This confirmed the presence of domain discrepancy in KOA KL grading. Direct merging of labeled source and target training samples provided a strong supervised adaptation baseline, which improved the QWK by 0.2184 and 0.2085 over source-only training in the K→M ResNet18 and $M_{\mathrm{aug}7}\to K$ ResNet18 settings, respectively.

Compared to direct merging, the proposed method achieved superior QWK scores across all four primary settings. In the K→M direction, our method demonstrated clear improvements over the Merge baseline for both backbones. These gains were further corroborated by improvements in MAE, Macro-F1, and BACC. Such results indicate that the proposed framework not only enhances the ordinal agreement but also reduces large-distance grading errors and boosts class-balanced sensitivity. In the reverse direction with the ResNet18, the QWK increased from 0.6782 to 0.7323, and the MAE decreased from 0.5978 to 0.5248. These results demonstrate that the proposed source sample screening and target-balanced ordinal contrastive learning modules remain highly effective and robust even when the adaptation direction changes.

The $M_{\mathrm{aug}7}\to K$ setting with VGG19 showed a boundary case of the proposed framework. In this setting, the proposed method obtained a marginally higher QWK than Merge, whereas Merge remained slightly better in ACC, MAE, Macro-F1, and BACC. Such results suggest that when the augmented MedicalExpert-I source set and the VGG19 backbone already provide a strong supervised adaptation baseline the additional contrastive constraint may offer limited extra benefit and slightly affect class-balanced performance. Therefore, the proposed method did not provide consistent improvements across all evaluation metrics in this configuration. This result differs from the clearer gains observed in the K→M settings and in the $M_{\mathrm{aug}7}\;\to \;K$ setting with ResNet18, indicating that the relative benefit of source-sample screening and ordinal contrastive alignment can vary across transfer directions and backbone architectures. In this configuration, the already strong performance of the supervised Merge baseline may leave a smaller margin for additional improvement.

#### 4.3.2. Comparison with Existing Methods

The benchmark comparison results in Table 4 and Table 5 demonstrate the effectiveness of the proposed framework compared with representative ordinal learning and domain adaptation approaches. Classical domain adaptation methods such as DANN improve cross-domain robustness by learning domain-invariant representations, but they do not explicitly consider the ordinal characteristics of KL grading. Similarly, ordinal learning approaches such as CORN and CLOC preserve severity ordering but lack mechanisms for reducing cross-domain distribution discrepancies.

**Table 4:** Comparison with representative methods on the K→M setting.

Method	QWK ↑	ACC ↑	MAE ↓	Macro-F1 ↑	BACC ↑
CORN [39]	0.8013	0.6126	0.4822	0.5663	0.5597
CLOC [36]	0.8414	0.6759	0.4032	0.6734	0.6828
DANN [31]	0.8860	0.7549	0.2964	0.7510	0.7627
SAT-DA [40]	0.9009	0.7391	0.2964	0.7466	0.7712
Ours	**0.9023**	**0.7945**	**0.2530**	**0.7958**	**0.7956**

Compared with these approaches, the proposed framework simultaneously considered domain discrepancy, ordinal relationships, and source-sample transferability. In particularly, compared with recent medical domain adaptation methods SAT-DA, our method achieved superior performance because it explicitly selected transferable source samples and introduced target-balanced ordinal contrastive alignment.

**Table 5:** Comparison with representative methods on the $M_{\mathrm{aug}7}\to K$ setting.

Method	QWK ↑	ACC ↑	MAE ↓	Macro-F1 ↑	BACC ↑
CORN [39]	0.7424	0.6002	0.5133	0.5796	0.5634
CLOC [36]	0.8156	0.6492	0.4106	0.6499	0.6550
DANN [31]	0.7472	0.5936	0.5224	0.5812	0.5842
SAT-DA [40]	0.7215	0.5791	0.5598	0.5238	0.5697
Ours	**0.8197**	**0.6624**	**0.3967**	**0.6593**	**0.6556**

The results indicate that simply aligning global feature distributions is insufficient for KOA grading under domain shift. Incorporating ordinal-aware and sample-selective adaptation strategies is more effective for maintaining clinically meaningful severity relationships across different radiographic domains.

#### 4.3.3. Confusion Matrix Analysis

**Figure 3:**
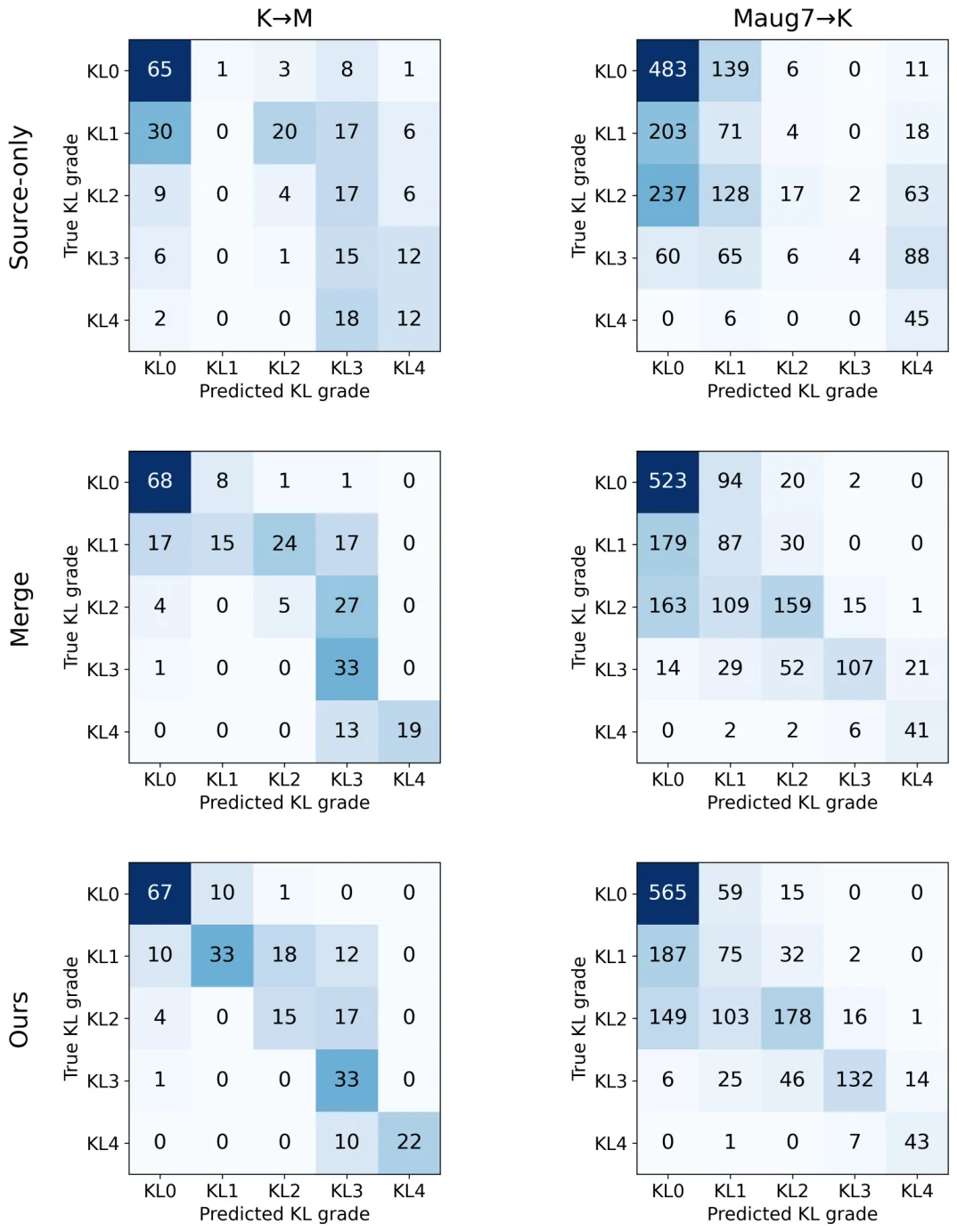
Confusion matrices of Source-only, Merge, and the proposed method using ResNet18. The first column shows the K→M setting, and the second column shows the $M_{\mathrm{aug}7}\;\to \;K$ setting. Each cell reports the corresponding number of test samples. The color intensity represents the number of samples in each cell, with darker colors indicating larger values.

To further examine the class-wise prediction behavior of different training strategies, confusion matrices were generated on the target-domain test sets for both transfer directions, as shown in Figure 3. ResNet18 was selected as the representative backbone for this analysis because it was also used in the ablation study and parameter-sensitivity experiments.

In the K→M setting, the Source-only model exhibited substantial confusion among the intermediate KL grades, particularly for KL1 and KL2. Direct source–target merging reduced this domain-induced degradation, whereas the proposed method further improved class-wise recognition. Compared with Merge, the number of correctly predicted KL1 samples increased from 15 to 33, KL2 samples increased from 5 to 15, and KL4 samples increased from 19 to 22, while the KL3 recognition remained stable (33 correctly predicted samples). In the reverse $M_{\mathrm{aug}7}\;\to \;K$ setting, the proposed method also improved several levels compared with Merge, with correctly predicted samples increasing from 523 to 565 for KL0, from 159 to 178 for KL2, from 107 to 132 for KL3, and from 41 to 43 for KL4. Although the number of correctly predicted KL1 samples decreased from 87 to 75, the overall performance still improved, with ACC increasing from 0.5537 to 0.5996 and QWK increasing from 0.6782 to 0.7323. These results indicate that the proposed framework improves recognition across multiple KL grades rather than benefiting only a specific severity category.

To further relate the confusion-matrix results to the ordinal nature of KL grading, we additionally analyzed prediction errors according to the absolute grade distance $|\hat{y}-y|$. In the K→M setting, the proposed method reduced both adjacent-grade errors ($|\hat{y}-y|=1$) and larger-distance errors ($|\hat{y}-y|\geq 2$) compared with Merge, decreasing the corresponding proportions from 35.2% to 25.7% and from 9.5% to 7.1%, respectively. Similarly, in the $M_{\mathrm{aug}7}\;\to \;K$ setting, adjacent-grade errors decreased from 30.6% to 28.0%, while larger-distance errors decreased from 14.1% to 12.0%. These findings provide an error-level explanation for the improvements in QWK and MAE, demonstrating that the proposed framework not only improves overall prediction accuracy but also reduces clinically undesirable large KL-grade deviations.

#### 4.3.4. Ablation Study

The ablation study on the ResNet18 backbone was implemented to investigate the distinct effects of the screening strategy and the contrastive module. The NoCF contrast applied the target-balanced ordinal contrastive learning module without source-domain sample screening. In contrast, the Plain filter setting employed the selected source samples but removed the proposed target-balanced ordinal weighting. The full method combined the selected source samples with the target-balanced ordinal contrastive learning module. In Table 6 and Table 7, “Proposed CL” denotes the target-balanced ordinal contrastive module, “Plain CL” denotes the standard supervised contrastive module, and “Sample screening” denotes the source-domain sample selection module. A checkmark (✓) indicates that the corresponding module is included in that configuration.

The ablation results first confirmed that direct source-target merging was a strong baseline, but it was still limited by treating all training samples in a uniform supervised manner. In both transfer directions, the NoCF contrast consistently outperformed the Merge baseline across all five metrics. These results demonstrated the effectiveness of the proposed target-balanced ordinal contrastive module even in the absence of source-domain sample screening. It indicates that incorporating KL-grade ordinality and target-domain class balance can help construct a more suitable cross-domain feature space for KOA grading.

**Table 6:** Ablation results for K→M with the ResNet18.

Method	Proposed CL	Plain CL	Sample Screening	QWK ↑	ACC ↑	MAE ↓	Macro-F1 ↑	BACC ↑
Merge				0.8089	0.5534	0.5494	0.5093	0.5561
NoCF contrast	✓			0.8300	0.6047	0.4822	0.5849	0.6209
Plain filter		✓	✓	0.8531	0.6561	0.4190	0.6384	0.6660
Ours	✓		✓	**0.8557**	**0.6719**	**0.4032**	**0.6545**	**0.6772**

**Table 7:** Ablation results for $M_{\mathrm{aug}7}\;\to \;K$ with the ResNet18.

Method	Proposed CL	Plain CL	Sample Screening	QWK ↑	ACC ↑	MAE ↓	Macro-F1 ↑	BACC ↑
Merge				0.6782	0.5537	0.5978	0.5489	0.5504
NoCF contrast	✓			0.7311	0.5972	0.5272	0.5931	0.5913
Plain filter		✓	✓	0.7135	0.5749	0.5537	0.5654	0.5671
Ours	✓		✓	**0.7323**	**0.5996**	**0.5248**	**0.5958**	**0.5942**

The comparison between NoCF contrast and Plain filter revealed the different roles of the two modules. In the K→M setting, the Plain filter clearly outperformed NoCF contrast, with an improvement of the QWK from 0.8300 to 0.8531 and a reduction of the MAE from 0.4822 to 0.4190. Such results indicate that filtering source samples with higher transfer value is particularly important when the source and target domains have a larger mismatch. In contrast, in the $M_{\mathrm{aug}7}\to K$ setting, the NoCF contrast performed better than the Plain filter, which demonstrated that the target-balanced ordinal contrastive learning module contributes more strongly when the augmented MedicalExpert-I set is used as the source domain.

Furthermore, the full proposed method achieved superior performance across both ResNet18 ablation directions. Compared with the Plain filter, our proposed approach further ameliorated the ACC, MAE, Macro-F1, and BACC results in the K→M setting, which indicated that the contrastive module was able to provide additional refinement after the source-sample screening. In the reverse setting, our method also slightly improved over both of the NoCF contrast and Plain filter, although the margin was smaller. These findings suggest that source-domain sample screening and target-balanced ordinal contrastive learning are complementary, with their relative contributions varying according to the transfer direction and the specific construction of the source domain.

#### 4.3.5. Parameter Sensitivity

**Figure 4:**
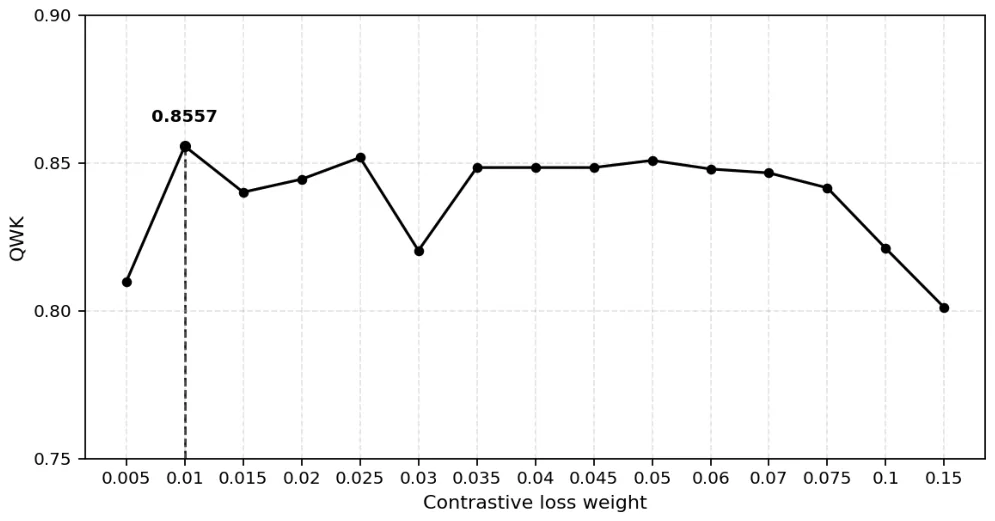
Sensitivity of QWK to $\lambda_{\mathrm{con}}$ for K→M with ResNet18.

**Figure 5:**
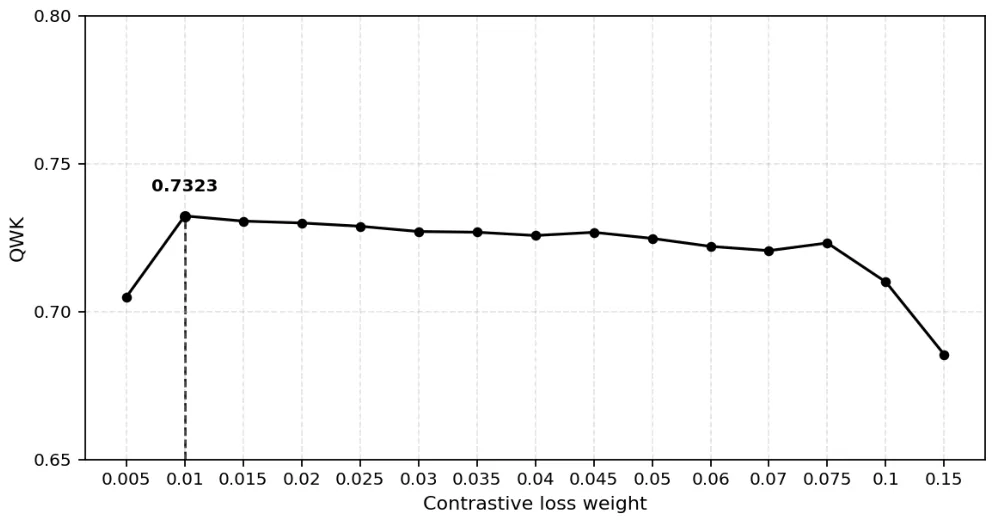
Sensitivity of QWK to $\lambda_{\mathrm{con}}$ for $M_{\mathrm{aug}7}\;\to \;K$ with ResNet18.

The parameter sensitivity analysis examined the contrastive loss weight $\lambda_{\mathrm{con}}$, which controlled the relative contribution of the contrastive objective to supervised classification. The analysis was performed with the ResNet18 in both transfer directions. Figure 4 and Figure 5 show the trends of the QWK results under different values of $\lambda_{\mathrm{con}}$, The dashed line marks the value of $\lambda_{\mathrm{con}}$ corresponding to the best QWK.

In the K→M setting, the model obtained a QWK of 0.8098 when $\lambda_{\mathrm{con}}=0.005$, which indicates that an excessively weak contrastive constraint is insufficient for effective feature alignment. The best QWK of 0.8557 was achieved at $\lambda_{\mathrm{con}}=0.01$. When the weight was further increased, the performance gradually decreased, with QWK dropping to 0.8210 at $\lambda_{\mathrm{con}}=0.1$.

A similar tendency could be observed in the $M_{\mathrm{aug}7}\;\to \;K$ setting. The best QWK was obtained at $\lambda_{\mathrm{con}}=0.01$, while larger weights led to a gradual performance decline. For example, QWK decreased from 0.7323 at $\lambda_{\mathrm{con}}=0.01$ to 0.7100 at $\lambda_{\mathrm{con}}=0.1$ and 0.6855 at $\lambda_{\mathrm{con}}=0.15$. These results indicate that contrastive loss should be used as an auxiliary feature-structure constraint rather than as a dominant optimization objective. A small contrastive weight can improve cross-domain feature organization, whereas an overly large weight may disturb the supervised KL decision boundaries.

#### 4.3.6. Feature Visualization

**Figure 6:**
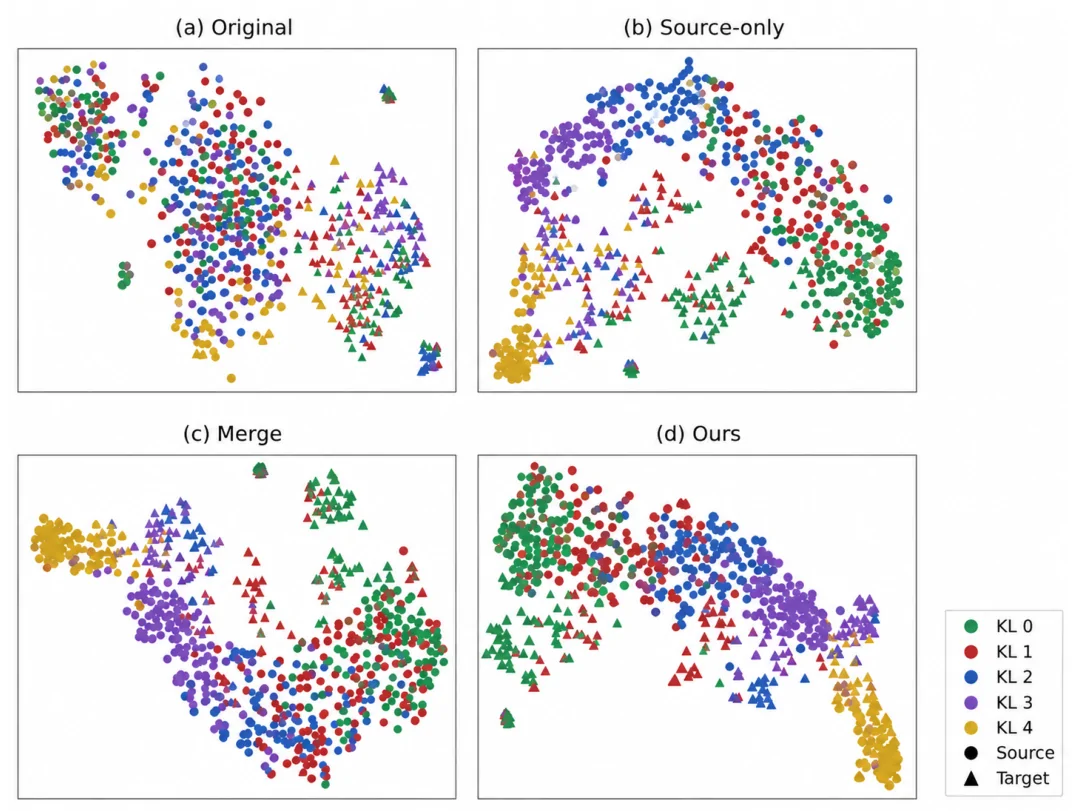
Visualizations of feature distributions with the t-SNE method under four training strategies. The four panels correspond to (**a**) the original ImageNet-pre-trained model, (**b**) source-only training, (**c**) direct source–target merging, and (**d**) the proposed method. Colors denote KL grades, and marker shapes denote source and target samples.

To qualitatively examine the learned feature space, t-SNE [41] was used to visualize features under four representative training strategies, including the original ImageNet-pre-trained model, source-only training, direct source–target merging, and the proposed method. The visualization was conducted in the K→M setting with the ResNet18. The visualization is shown in Figure 6.

The original ImageNet-pretrained model showed weak grade-related organization, as it had not been optimized for the specific task of KOA grading. Source-only training improved the class layout within the source domain but still presented limited cross-domain consistency. Direct merging provided better target-domain organization by using labeled target samples during training. Compared with these training strategies, the full model yielded a clearer grade-wise feature organization, with more compact same-grade samples and smoother transitions between adjacent KL grades. This qualitative observation was consistent with the quantitative improvements observed in the corresponding experimental settings.

## 5. Discussion

The experimental results first demonstrated the importance of supervised domain adaptation for KOA KL grading when compared with the internal training baselines. Source-only training performed poorly in both transfer directions and with both CNN backbones, which indicates that models trained on a single radiograph dataset fail to generalize effectively to a new dataset without adaptation. Direct merging substantially improved performance and therefore served as a strong baseline. The full proposed model further surpassed this baseline across all evaluated settings on the primary QWK metric. Such results indicate that the performance gains were not merely attributable to the inclusion of labeled target data but also benefited from more selective source utilization and superior cross-domain feature organization.

Beyond these internal baselines, the comparisons with representative existing methods further demonstrated the competitiveness of the proposed framework. Across the two transfer directions, the proposed framework achieved the highest QWK among the compared existing methods, including 0.9023 in the K→M setting and 0.8197 in the $M_{\mathrm{aug}7}\;\to \;K$ setting. These results suggest that jointly considering source-sample transferability, cross-domain discrepancy, and ordinal severity relationships provides a complementary advantage over approaches that focus primarily on either domain alignment or ordinal modeling alone.

The ablation study clarified the distinct contributions of two key components. Source-domain sample screening is vital, as not all source samples contribute equally to target-domain adaptation. In the KOA grading, adjacent KL grades were visually similar, and target-inconsistent source samples may have introduced misleading contrastive relations. Selecting source samples with higher transfer value reduced this risk and provided a stronger basis for cross-domain alignment. On top of this selected source set, the target-balanced ordinal contrastive loss further introduced the KL-grade distance and target-domain class-frequency information, which was consistent with the ordinal and imbalanced nature of KOA grading.

The results also show that the contribution of each component was not identical across transfer directions and backbones. In the K→M direction, the proposed method consistently provided clear improvements over Merge, especially with VGG19. In the reverse direction, the ResNet18 setting still benefited from the full framework, whereas the VGG19 setting showed only a marginal QWK gain over Merge, with Merge remaining slightly better in ACC, MAE, Macro-F1, and BACC. This observation is important for a fair interpretation of the method: the contrastive constraint is helpful in most settings, but its effectiveness can depend on the source-domain construction, target-domain distribution, and backbone capacity.

The parameter sensitivity results suggested that contrastive learning should be treated as an auxiliary regularization term: a moderate value of $\lambda_{\mathrm{con}}$ improved the feature alignment, but excessive contrastive weighting reduced the performance. This is reasonable for KL grading because adjacent grades have inherent visual continuity. If the contrastive objective becomes too strong then it may overemphasize global feature separation and weaken the classification head’s ability to fit fine-grained grade boundaries.

Several limitations of the present study also indicate directions for future research. First, because the current framework relies on labeled target-domain samples, future work will investigate semi-supervised and unsupervised adaptation to reduce the dependence on target-domain annotations. Second, the framework should be further validated on additional external and multicenter datasets and with more diverse encoder architectures to assess its robustness across heterogeneous clinical settings. Third, the current source-sample screening strategy uses predefined source-retention and core/adapt ratios; future work will explore adaptive sample-selection mechanisms driven by domain discrepancy, class difficulty, or predictive uncertainty. Finally, incorporating uncertainty estimation and model calibration may improve the reliability of predictions for ambiguous cases near adjacent KL-grade boundaries.

## 6. Conclusions

The present study proposes a supervised domain adaptation framework for five-class radiographic KOA KL grading. To mitigate negative transfer induced by domain shift, the proposed method screens source-domain samples according to transfer difficulty and prototype-based sample quality, and it then aligns them with the target-domain class structure through target-balanced ordinal contrastive learning. By jointly integrating source-sample transfer value, target-domain class balance, and KL-grade ordinality, the framework learns a more discriminative and clinically consistent feature space.

Extensive experiments on the KneeKL and MedicalExpert-I datasets demonstrated that the proposed method consistently outperformed source-only training and achieved superior QWK performance over direct source–target merging across all settings. Comparisons with representative existing methods further supported the competitiveness of the proposed framework, while confusion-matrix and ordinal error analyses showed improved class-wise recognition and fewer large KL-grade deviations. Furthermore, the ablation results confirmed the complementary effects of source-domain sample screening and target-balanced ordinal contrastive learning for robust KOA severity grading under domain shift.

## Figures and Tables

**Table 1:** Class distribution of the two knee radiograph datasets used in the experiments.

Dataset	Split	Total	KL0	KL1	KL2	KL3	KL4
	Train	5778	2286	1046	1516	757	173
KneeKL	Val	826	328	153	212	106	27
	Test	1656	639	296	447	223	51
	Train	1152	359	333	162	154	144
MedicalExpert-I	Val	245	77	71	34	33	30
	Test	253	78	73	36	34	32

**Table 2:** Performance results for K→M with ResNet18 and VGG19 on the target-domain testing set.

Backbone	Method	QWK ↑	ACC ↑	MAE ↓	Macro-F1 ↑	BACC ↑
ResNet18	Source-only	0.5905	0.3794	0.9526	0.2865	0.3521
	Merge	0.8089	0.5534	0.5494	0.5093	0.5561
	Ours	**0.8557**	**0.6719**	**0.4032**	**0.6545**	**0.6772**
VGG19	Source-only	0.4287	0.2727	1.0949	0.2728	0.3459
	Merge	0.8728	0.7787	0.2925	0.7693	0.7598
	Ours	**0.9023**	**0.7945**	**0.2530**	**0.7958**	**0.7956**

**Table 3:** Performance results for $M_{\mathrm{aug}7}\;\to \;K$ with ResNet18 and VGG19 on the target-domain testing set.

Backbone	Method	QWK ↑	ACC ↑	MAE ↓	Macro-F1 ↑	BACC ↑
ResNet18	Source-only	0.4697	0.3744	0.9710	0.2458	0.3868
	Merge	0.6782	0.5537	0.5978	0.5489	0.5504
	Ours	**0.7323**	**0.5996**	**0.5248**	**0.5958**	**0.5942**
VGG19	Source-only	0.4534	0.3406	0.8653	0.3294	0.4190
	Merge	0.8191	**0.6643**	**0.3961**	**0.6639**	**0.6623**
	Ours	**0.8197**	0.6624	0.3967	0.6593	0.6556

## Data Availability

The datasets used in this study are publicly available at https://data.mendeley.com/datasets/56rmx5bjcr/1 [16] (accessed on 22 August 2026) and https://data.mendeley.com/datasets/t9ndx37v5h/1 [17] (accessed on 22 August 2026).
